# Journals’ instructions to authors: A cross-sectional study across scientific disciplines

**DOI:** 10.1371/journal.pone.0222157

**Published:** 2019-09-05

**Authors:** Mario Malički, IJsbrand Jan Aalbersberg, Lex Bouter, Gerben ter Riet

**Affiliations:** 1 Amsterdam UMC, University of Amsterdam Department of Cardiology, Academic Medical Center, Amsterdam, the Netherlands; 2 ACHIEVE Centre for Applied Research, Faculty of Health, Amsterdam University of Applied Sciences, Amsterdam, the Netherlands; 3 Elsevier, Amsterdam, the Netherlands; 4 Amsterdam UMC, Department of Epidemiology and Biostatistics, VU University Medical Center, Amsterdam, the Netherlands; 5 Department of Philosophy, Faculty of Humanities, Vrije Universiteit, Amsterdam, the Netherlands; Central South University, The Third Xiang Ya Hospital, CHINA

## Abstract

In light of increasing calls for transparent reporting of research and prevention of detrimental research practices, we conducted a cross-sectional machine-assisted analysis of a representative sample of scientific journals’ instructions to authors (ItAs) across all disciplines. We investigated addressing of 19 topics related to transparency in reporting and research integrity. Only three topics were addressed in more than one third of ItAs: conflicts of interest, plagiarism, and the type of peer review the journal employs. Health and Life Sciences journals, journals published by medium or large publishers, and journals registered in the *Directory of Open Access Journals (DOAJ)* were more likely to address many of the analysed topics, while Arts & Humanities journals were least likely to do so. Despite the recent calls for transparency and integrity in research, our analysis shows that most scientific journals need to update their ItAs to align them with practices which prevent detrimental research practices and ensure transparent reporting of research.

## Introduction

Since its origin in the 17th century, scientific publishing has gone through many changes. From unstructured abstracts and manuscript formats to formal structuring,[1] increase in the number of authors and shared (first or last) authorship,[2] from paper-based to predominantly online content,[3] development of different payment and distribution methods,[3] as well as different methods of impact measurement of articles and journals.[4] Lately, there has also been a drive towards prospective study registration[5, 6] publishing of manuscripts on pre-print servers before they are peer-reviewed,[7] use of reporting guidelines to address the completeness of reporting,[8] data sharing, conducting replication studies,[9] and more emphasis on post-publication peer review.[10] Many of the latter initiatives have also been introduced to foster responsible conduct of research.[11] However, these practices are neither harmonized across scientific disciplines, nor globally enforced. Studies have shown that detrimental research practices still stain scientific publishing,[12] with up to 50 percent of conducted studies not being published,[13, 14] main outcome measures listed in study protocols being changed in publications of results,[13, 15] unexpected findings or results being reported as having been hypothesised during study design,[16–18] and the improper statistical methods being used in analyses.[19] Journals or editors have often been portrayed as gatekeepers against these practices, with journal’s instructions to authors (ItAs), documents meant to help authors prepare their manuscripts for submissions, also being used to raise awareness of these issues. In parallel with this study, we also conducted a systematic review of studies that analysed ItAs and identified 153 studies assessing more than 100 topics (not)covered in ItAs. However, as none of those studies aimed to compare differences between scientific disciplines using a representative sample of journals across all journal impact factors,[20] we sought to learn what journals from different scientific disciplines recommend or demand in their instructions to authors regarding transparency in reporting and research integrity topics.

## Materials and methods

A detailed methods description is available as a published study protocol on our projects’ data repository site.[21] In short, we conducted a machine assisted cross-sectional analysis of 835 journals’ ItAs, downloaded from journals’ websites between 14 December 2017 and 24 January 2018. The number of journals for analysis was pre-calculated to represent all journals classified in *Scopus* as exclusively belonging to one of the following scientific disciplines: Arts & Humanities, Health Sciences, Life Sciences, Physical Sciences, or Social Sciences (N = 14,708). Proportional numbers of journals from all terciles of *Source Normalized Impact per Paper (SNIP)* and scientific disciplines were sampled. Additionally, we analysed all available ItAs of journals classified as multidisciplinary in either *Scopus* or *Science Citation Index Expanded*–Multidisciplinary Sciences category (N = 94). For questions regarding ItAs or obtaining their English versions, we contacted 125 journals, and received responses from 38 (30%).

### Variables

From the *Scopus Source List,[22]* for each journal we extracted:

1. Journal’s *SNIP* value for 2016 (numerical variable).
2. Journal’s publisher (nominal variable)—we further categorised the publishers into 3 groups: a) large publishers: *Taylor & Francis*, *Elsevier*, *Springer Nature*, and *Wiley-Blackwell*, each having 66 to 72 journals in our sample; b) medium publishers, those publishing 2–22 journals in our sample; and c) small publishers, those with only one journal in our sample.
3. Journal publisher’s country (nominal variable).
4. Journal’s indexation in the *Directory of Open Access Journals (DOAJ)* database (binary variable).

### Topic selection

**Fig 1:**
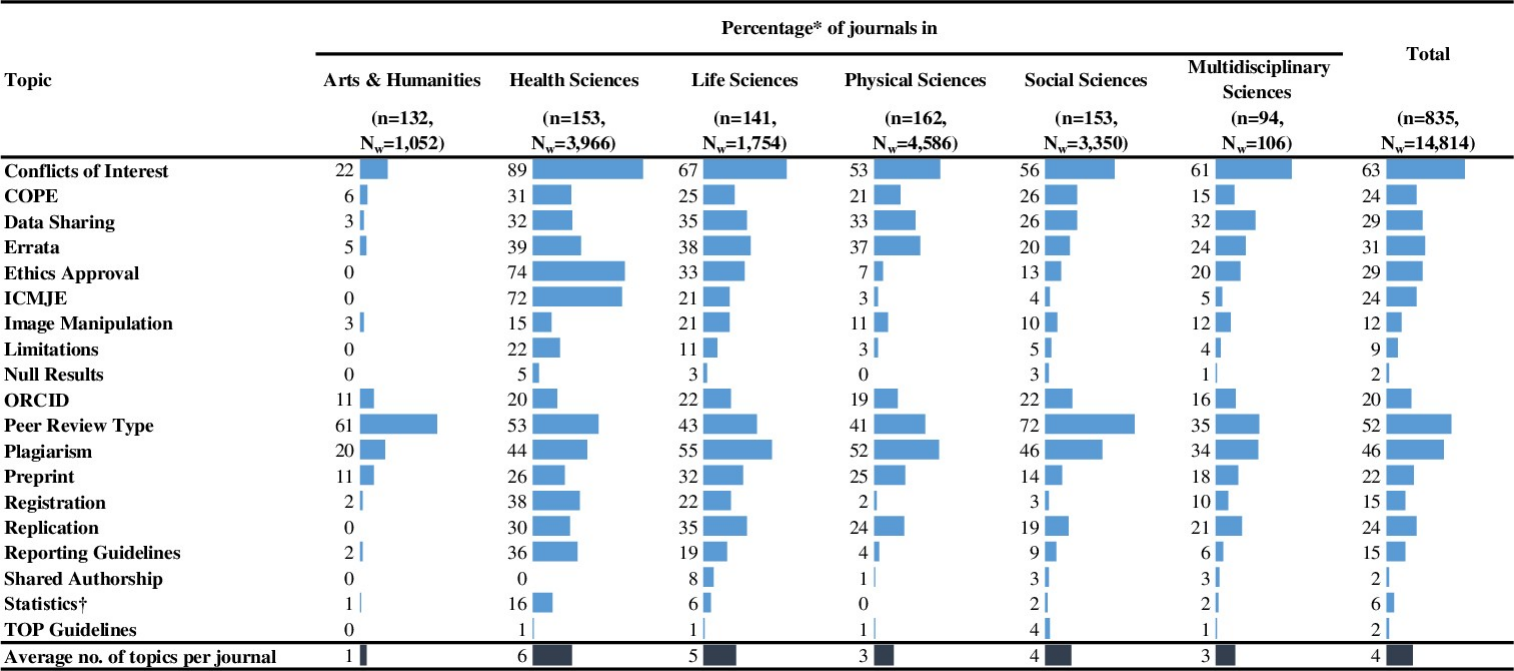
Percentages of journals covering transparency in reporting and research integrity topics in their instructions to authors. *Our sample size was 835 journals (n). All analyses were performed in STATA (version 13) using sampling weights representing a total of 14,814 journals (Nw). † Addressing at least one of the following topics: Bayesian statistics, confidence intervals, sample size, and effect size.

Following consultations with project advisors (listed in the acknowledgments) and based on results of our systematic review of studies that analysed instructions to authors,[23] we selected 19 topics on transparency in reporting and research integrity (ordered alphabetically and described in detail below). Each topic was described with at least two variables: a) a binary variable–indicating whether the topic is or is not mentioned in the ItAs (shown in the results in Fig 1 and S1 Table); b) a nominal variable–indicating *how* the topic was mentioned, e.g. recommended or required, or described using specific wording (shown in results section under each topic’s subheading). For four topics (specified below), we also checked how they were addressed in journals’ scope statements using the same classification system. The 19 topics were:

1. Conflicts of Interest: We checked if the words *conflict*, *competing* or *declaration of interest*(*s*) were mentioned, or whether authors were asked to declare funding, financial or grant details. Additionally, we checked if *Crossref Funder Registry* was recommended for correct nomenclature of funding bodies.[24]
2. COPE: We checked if *Committee on Publication Ethics (COPE)* was mentioned or recommended to authors. This topic was also checked in journals’ scope statements.
3. Data Sharing: We checked if ItAs recommended or required data sharing in general, or for specific types of data (e.g. depositing of DNA sequences in genetic databases or X-ray crystallographic structures in crystallographic databases), or if data(sets) could be accepted as supplementary materials. We also checked mentioning of *Dryad*, *Figshare* and the *Registry of Research Data Repositories (Re3data*.*org)*.
4. Errata: We checked if corrections of papers after publication (i.e. errata or corrigenda) were mentioned, and if they were mentioned only in specific instances (e.g. detection of image manipulation, changes in authorship or undisclosed conflicts of interest).
5. Ethics Approval: We checked if reporting of ethics approval was required, or if studies needed to be conducted according to the *Declaration of Helsinki* (any version).
6. ICMJE: We checked if *International Committee of Medical Journal Editors (ICMJE)* were referred to for any of their recommendations (e.g. manuscript formatting, trial registration, authorship definition, conflicts of interest, statistical guidance).
7. Image Manipulation: We checked if (screening for) image manipulation or duplication was mentioned.
8. Limitations: We checked if study limitations should be addressed anywhere in the manuscript.
9. Null Results: We checked if studies with null or negative results would be considered for publication. This topic was also checked in journals’ scope statements.
10. ORCID: We checked if an *Open Researcher and Contributor ID (ORCID)* was recommended or required from authors.
11. Peer Review Type: We checked if the type of peer review the journal uses (i.e. open, single, double or triple blind) was mentioned. We classified anonymous peer review and blinded peer review as single blind, unless explicitly described as double or triple anonymous. This topic was also checked in journals’ scope statements.
12. Plagiarism: We checked if (screening for) plagiarism was mentioned and (if) the service or software used was specified.
13. Preprint: We checked if posting or archiving manuscripts on personal websites or pre-prints before submission to the journal were (dis)allowed.
14. Registration: We checked if studies, materials or protocols needed to be registered before manuscript submission.
15. Replication: We checked if publication of replication studies was mentioned or if methods and analysis sections should be written in ways to facilitate replication. This topic was also checked in journals’ scope statements.
16. Reporting Guidelines: We checked if reporting guidelines were required or recommended (we coded expressions such as *must* or *require* as a requirement; words like *(strongly) recommend*, *may*, or *encourage* were coded as *recommend*). Additionally, we checked if the ItAs mentioned the following specific guidelines: *Animal Research*: *Reporting of In Vivo Experiments (ARRIVE)*, *Consolidated Standards of Reporting Trials (CONSORT)*, *Preferred Reporting Items for Systematic Reviews and Meta Analyses (PRISMA)*, *Strengthening the Reporting of Observational Studies in Epidemiology (STROBE)*, or the *Enhancing the QUAlity and Transparency Of health Research Network (EQUATOR)*.
17. Shared Authorship: We checked if authors could declare to have equally contributed to the manuscript, either as equal first or last authors, and if the number of such authors was limited.
18. Statistics: We checked whether reporting Bayes factor, confidence intervals, effect sizes, power or sample size calculations was required or recommended.
19. TOP Guidelines: We checked whether *Transparency and Openness Promotion (TOP)* guidelines were mentioned.

### Topic data extraction

The addressing of the above-mentioned topics in the ItAs or scope statements was checked by constructing regular expressions (search scripts) using keywords for each topic. We used *Practical Extraction and Reporting Language (PERL*, *Strawberry Perl for Windows)* for parsing the ItAs into sentences (using *Lingua*::*EN*::*Sentence* module)[25] and for searching those sentences with the regular expressions. All *PERL* scripts used were constructed and edited using *Notepad++*. All extracted matching sentences and regular expression were then checked by MMal and coded as described in the variables section above. Full text of ItAs was checked in case the sentences were ambiguous. To check that all ItAs were properly stored as text files (*Windows* character *ANSI* encoded), and that the sentence extraction and regular expressions worked as intended, we ran scripts for words we expected to find in all ItAs, namely: a) *article* or *manuscript*, b) *author*, and c) *reference* or *literature*. Out of the 835 ItAs in our sample, positive matches were obtained for 829, 829, and 755 journals, respectively, with all 835 ItAs containing at least one of the 5 words. Additionally, to test for a word that is expected to be much more prevalent in one scientific discipline than in others, we tested our method on mentioning of (acceptance of) *LaTex* files for manuscript submission (expected to be most prevalent in Physical Sciences (n = 103, 64% in Physical Sciences *vs* n = 18–52, 14–48% in other Sciences, S1 Table). As a final check that the keywords and the regular expressions we constructed did not fail to detect the topics we were interested in, on 26 July 2018, after all data for all topics were checked, MMal read full ItAs of 24 (27%) out of 88 journals that showed no results for any of the topics. The 88 ItAs without any topic matches were from all 6 disciplines (28 from Arts & Humanities, 5 from Health Sciences, 11 from Life Sciences, 14 from Physical Sciences, 16 from Social Sciences, and 14 for Multidisciplinary Sciences), and so we randomly sampled 4 from each discipline using the same random number generator as described in our protocol for selection of the journals.[23] Reading the full ItAs, we found that two contained information on accepting *tex* files for publication instead of *LaTeX*, and one journal’s ItA misspelt the full name of *ICMJE* and did not use *ICMJE* as an acronym. We therefore concluded that no adjustments were needed for the scripts for the topics.

### Statistical analysis

We conducted all analyses in *STATA* v.13, using the survey setting, with sampling weights calculated as the total number of journals from the *All Science Journal Classification (ASJC)* tercile the journal was sampled from (N_w_), divided by the number of journals sampled from that tercile (n), while finite population correction and the survey strata were based on the number of journals in the corresponding *ASJC* fields. All percentages, odds ratios and confidence intervals reported are based on the weighted analyses as described above (based on the total number of 14,814 journals; details available on our projects’ data depository site).[23] All percentages are rounded to the full number, except percentages lower than 1, which are rounded to one decimal place.

Logistic regression was used to explore to which extent *SNIP*, registration in the *DOAJ*, 3 categories of publishers, and the six scientific disciplines, were associated with the likelihood of the topics being mentioned (reference categories for the regression analyses were: 1) *SNIP* increase of 1 unit; 2) not registered in the *DOAJ;* 3) belonging to small publishers (defined as having only 1 journal in our sample from the same publisher); and 4) Multidisciplinary Sciences journals. The logistic regression model contained all above-mentioned determinants. As stated in our protocol,[21] we chose these factors as previous research has indicated their association with mentioning of specific topics in ItAs.[26, 27] Additionally, as we based our sample on journals indexed in Scopus, DOAJ registration (a proxy for open access publishing model). publisher information, and SNIP values (a citation metric adjusted to allow for direct comparison between different scientific fields),[28] were all available directly from the *Scopus Source List* which we used for journal sampling.[22]

### Data sharing

All data, scripts with regular expressions, generated random numbers and their matching journals, alongside data extraction notes, are available on our project’s data repository site.[23]

## Results

### Journal sample description

In total we obtained 835 journal ItAs, and 817 (98%) journal scope statements. The journals belonged to 420 different publishers, specifically 370 (44%) belonged to small publishers (with no other journals in our sample), 189 (23%) to medium publishers (with a median of 3 journals in our sample, range 2–20), and 276 (33%) to the 4 major publishers: *Taylor & Francis* (n = 72, 9%), *Elsevier* (n = 72, 9%), *Springer Nature* (n = 66, 8%), and *Wiley-Blackwell* (n = 66, 8%). Publishers were located in 66 different countries, most commonly *USA* (n = 210, 25%), *UK* (n = 186, 22%), *the Netherlands* (n = 67, 8%), *Germany* (n = 49, 6%) and *India* (n = 31, 4%). (Note: Different journals from the same publisher can be from different countries). Lastly, 163 (20%) of journals were registered in the *DOAJ*.

**Fig 2:**
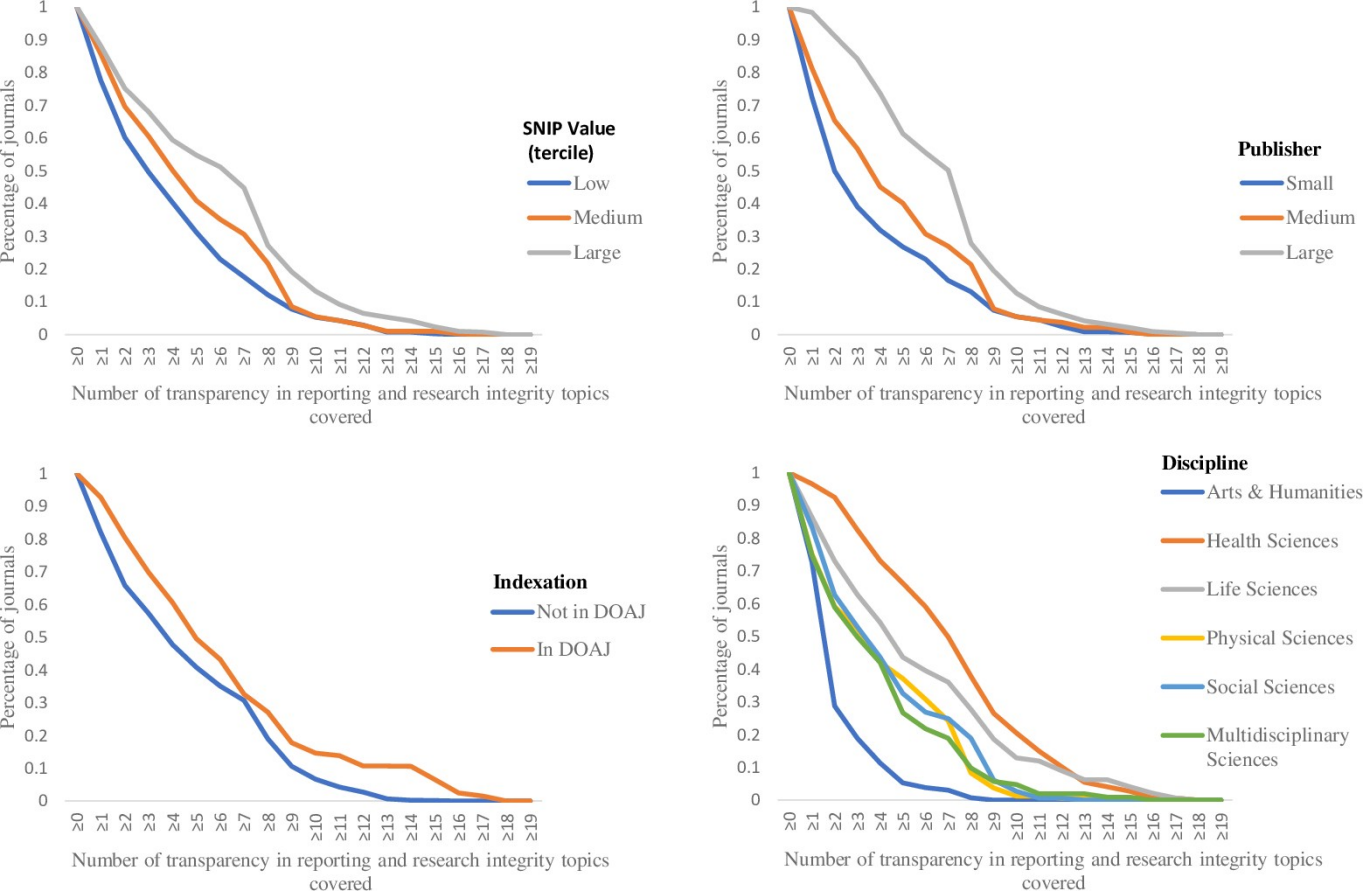
Differences in percentages of journals addressing transparency in reporting and research integrity topics according to Source Normalized Impact per Paper (SNIP) terciles; publisher size (large: Taylor & Francis, Elsevier, Springer Nature, and Wiley-Blackwell; medium: those with 2–22 journals in our sample; and small: those with only 1 journal in our sample); registration in Directory of Open Access Journals (DOAJ) database; and scientific discipline.

Only two of the topics that we checked in ItAs (Conflicts of Interest and Peer Review Type) were addressed in more than half of ItAs (63% and 52%, respectively), and one, Plagiarism, in more than one third of ItAs (46%), while the remaining 16 topics were mentioned in less than one third of ItAs (0% to 31%) (Fig 1, S1 Table). The (weighted) average number of topics addressed per journal across all disciplines was 4 (95%CI 4–5), however it was lowest in Arts & Humanities journals (M_w_ = 1, 95%CI_w_ 1–2), and highest in Health Sciences journals (M_w_ = 6, 95%CI_w_ 6–7). Furthermore, differences (from lowest to highest) in the (average) number of topics were also observed between small, medium and large publishers, journals not registered and registered in the *DOAJ*, and journals with low, medium and high *SNIP* values (Fig 2).

**Fig 3:**
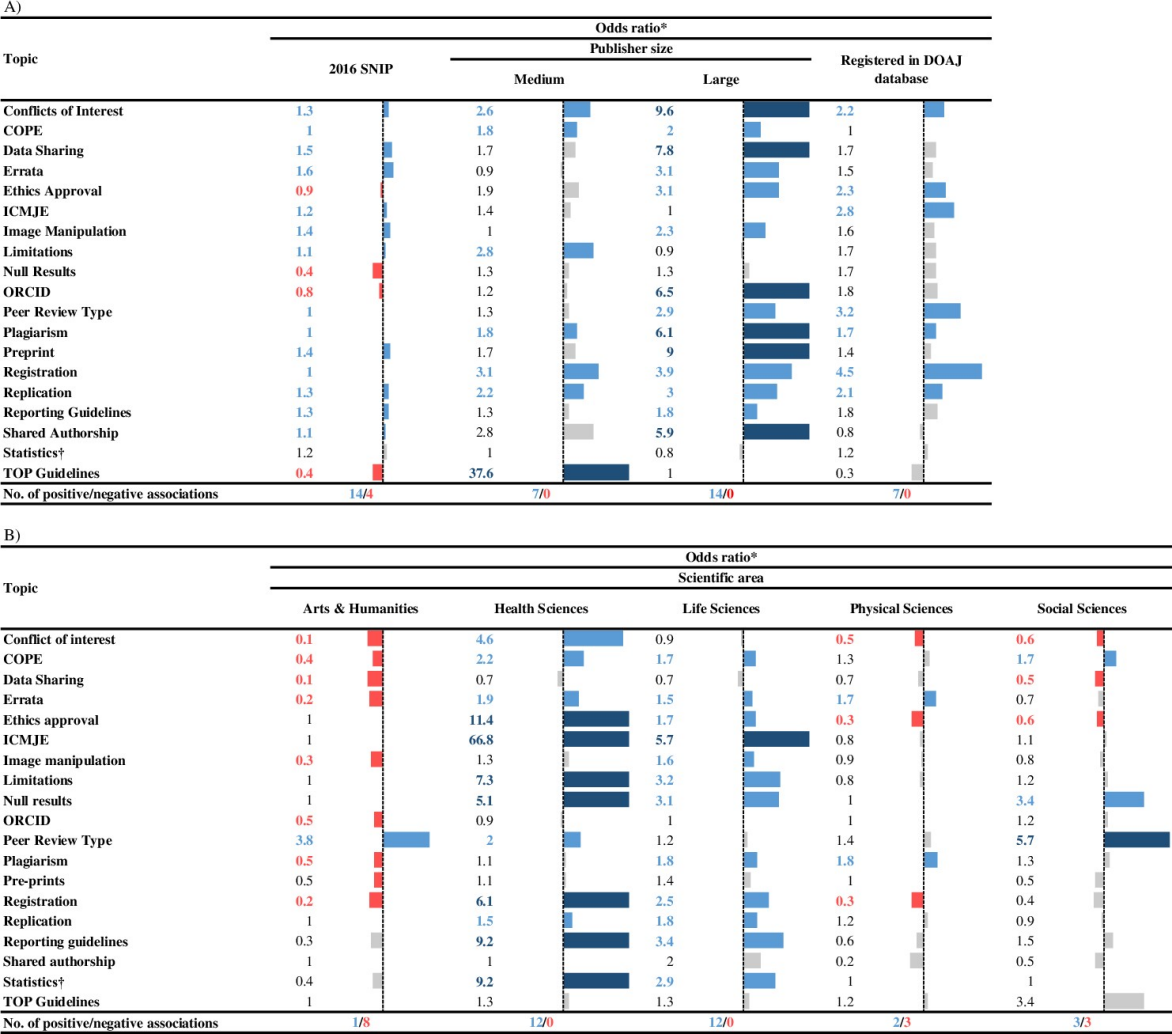
**Association (odds ratios from regression analysis) of transparency in reporting and research integrity topics addressed in instructions to authors of journals with: A) Source Normalised Impact per Paper (SNIP) values, registration in the Directory of Open Access Journals (DOAJ), publishers’ category (medium or large sized publishers) and; B) top scientific areas. Red and light blue numbers and bars indicate statistically significant associations, while grey ones indicate statistically non-significant associations. Dark blue bars and numbers indicate odds ratios higher than 5. All odds ratios are rounded to one decimal place.** *Our sample size was 835 journals (n). All analyses were performed in STATA (version 13) using sampling weights representing a total of 14,814 journals (Nw). For the regression analyses, reference categories were: 1) SNIP increase of 1; 2) Not registered in DOAJ; 3) Belonging to small publishers (defined as having only 1 journal in our sample form the same publisher); 4) Multidisciplinary Sciences journals.

In the regression analyses, independent associations for all factors we explored (journal’s *SNIP* value, publisher size, registration in the *DOAJ*, or scientific disciplines were confirmed (Fig 3, S2 Table). Details of these analyses are described in the topic-specific subsections below.

### Topics

1. Conflicts of Interest: Some type of declaration of conflicts of interest (COI) was required by 63% of journals; 9% required authors to declare funding or grant(s) associated with the study, but did not use the words *conflict*, *competing* or *declaration of interest* (which were used by 4%, 29% and 1% of journals, respectively), with an additional 18% of journals using both *conflict of* and *competing interest* in their ItAs. In our sample, an interesting phrasing was found in 16 journals (9 from *Springer Nature*) stating authors should declare everything that could *“embarrass”* them *“were they to become publicly known after the work was published*.*”* 10 journals in our sample mentioned the *Crossref Funder Registry* as a way for authors to check for the correct nomenclature of the funders, 8 of which were published by *Wiley-Blackwell*.
  ItAs of journals belonging to Health Sciences, or published by medium or large publishers, or registered in the *DOAJ*, were more likely, while those belonging to Arts & Humanities, Physical Sciences, and Social Sciences were less likely to mention conflicts of interest.
2. COPE: COPE was mentioned in 24% of ItAs, and in an additional 1% in the journals’ scope statements. ItAs of journals belonging to Health Sciences, Life Sciences, and Social Sciences, or published by medium or large publishers, were more likely, while those belonging to Arts & Humanities were less likely to mention COPE.
3. Data Sharing: Data sharing was mentioned in 29% of ItAs, of which all recommended it except 0.8% that required it. Additionally, 11% accepted data(sets) as supplementary materials, while 1% recommended and 1% required only specific data to be shared (e.g. DNA sequences in genetic databases or X-ray crystallographic structures in crystallographic databases). In regards to specific repositories 5% of ItAs recommended the *Dryad* repository, 11% *Figshare*, and 1% directed the authors to check the *Registry of Research Data Repositories (Re3data*.*org)* for an appropriate repository.
  ItAs of journals with higher *SNIP* values, or published by large publishers, were more likely, while those belonging to Arts & Humanities, and Social Sciences were less likely to mention data sharing.
  As for the data reported within the study, 3 journals in our sample, 2 published by *Wolters Kluwer Health* and one by *Springer Nature*, required authors to declare during manuscript submission that *“all the data collected during the study is presented in this manuscript and no data from the study has been or will be published separately”*.
4. Errata: Correcting papers post-publication was mentioned in 31% of ItAs: 21% by publishing errata or corrigenda, 1% as letters to editors, while the remaining mentioned corrections only in specific instances (10% for changes in authorship. 1% for image manipulation, and 1% for undisclosed conflicts of interest).
  ItAs of journals belonging to Health Sciences, Life Sciences, and Physical Sciences, those with higher *SNIP* values, or published by large publishers, or registered in the *DOAJ*, were more likely, while those belonging to Arts & Humanities were less likely to mention errata.
5. Ethics Approval: Ethics approval was mentioned in 29% of ItAs: 7% requiring institutional or ethics review board approval, 3% that the study is conducted according to the *Declaration of Helsinki*, while 20% mentioned both.
  ItAs of journals belonging to Health Sciences and Life Sciences, or published by large publishers, or registered in the *DOAJ*, were more likely, while those belonging to Physical Sciences and Social Sciences were less likely to mention ethics approval.
6. ICMJE: *ICMJE* was mentioned by 24% of journals, with one journal from the Health Sciences using full *ICMJE*’s *Recommendations for the Conduct*, *Reporting*, *Editing*, *and Publication of Scholarly work in Medical Journals* as their ItA.
  ItAs of journals belonging to Health Sciences and Life sciences, or those registered in the *DOAJ*, were more likely to mention *ICMJE*.
7. Image Manipulation: Prohibition of image manipulation was mentioned in 12% of ItAs, while 2% stated they would screen all images for manipulation upon manuscript submission.
  ItAs of journals belonging to Life Sciences, or those with higher *SNIP* values, or published by large publishers, or registered in the *DOAJ*, were more likely, while those belonging to Arts & Humanities were less likely to mention image manipulation.
8. Limitations: Reporting of studies limitations was mentioned in 9% of ItAs; with only 1% requiring a limitations section. ItAs of journals belonging to Health Sciences and Life Sciences, or those published by medium publishers, were more likely to mention study limitations.
9. Null Results: Only 1% of ItAs stated that studies with null or negative results will be considered for publication, while an additional 1% stated they can be published as short papers. ItAs of journals belonging to Health Sciences, Life Sciences, and Social Sciences were more likely to mention studies with null or negative results. Only 4 journals mentioned accepting null or negative results in their scope statements, 3 of which also did so in their ItAs.
10. ORCID: ItAs of 16% of journals recommended authors to list their *ORCID* during manuscript submissions, while 4% required it. ItAs of journals published by large publishers were more likely, while those belonging to Arts & Humanities were less likely to mention *ORCID*.
11. Peer Review Type: ItAs of 52% of journals mentioned the peer review type that the journal used, with 26% stated they use single blind, 26% double blind, 0.1% stating triple blind, and 0.1% allowing the authors to choose between single and double blind. Only Multidisciplinary journals (2%) mentioned using open peer review. One journal, alongside single blind peer review that they normally used, described an option of *revisionless* peer review, that allows “*senior*, *established*” authors to have their manuscripts published as submitted, if the reviewers find it acceptable for publication, without the need to address reviewers’ or editor’s comments (the authors can, but are not obliged to, address the comments). Two journals addressed the peer review cost in their ItAs, one stating that if authors withdraw a paper after it has passed peer review or typesetting they would be charged US$50 for the “peer review and typesetting cost”, while the other stated that if authors ask for a rapid evaluation of their manuscript they will need to leave a deposit of 200 Euro, which if the manuscript is deemed unsuitable for publication, will not be eligible for a refund. We also searched for descriptions of peer review type in journals’ scope statements, and found 87 (11%) of journals stated the peer review type, but all that did so, also stated that information in their ItAs. Finally, in our sample, 2 journals encouraged authors to have their manuscripts reviewed by colleagues before they submit them to the journal, and one journal stated that because inferior or flawed methods are the most common reasons behind manuscript rejection, authors should have the designs of their studies peer reviewed before starting the data collection.
  ItAs of journals belonging to Arts & Humanities, Health Sciences and Social Sciences, or published by large publishers, or registered in the *DOAJ*, were more likely to mention the peer review type.
12. Plagiarism: Plagiarism was addressed in 46% of ItAs: 19% stating that all manuscripts submitted to the journal will be screened for plagiarism (most commonly using the *iThenticate* software, 14%), 17% stating that the manuscripts may be checked for plagiarism, and 10% addressing plagiarism, but not specifying if they will screen for it. In our sample 4 ItAs addressed the amount of similarity acceptable within manuscripts; 3 journals, all published by *Bentham Science Publishers*, stated that the similarity index should be less than 20%, with a maximum of 15% of similar text taken from a single article, while one journal stated that the similarity index should be less than 15%, with a maximum of 5% taken from a single source.
  ItAs of journals belonging to Life Sciences and Physical Sciences, or published by medium or large publishers, or registered in the *DOAJ*, were more likely, while those belonging to Arts & Humanities were less likely to mention plagiarism.
13. Preprint: ItAs of 22% of journals mentioned that manuscripts deposited on preprint servers or self-archived before being submitted to the journal will be accepted for peer review and publication, while 1% of journals explicitly stated they would not be. Additionally, 1% of journals asked authors not to post the revised version on the preprint server.
  ItAs of journals published by large publishers were more likely to mention preprints.
14. Registration: Study, material or protocol registration was addressed in 15% of ItAs: 10% requiring studies, and 0.1% requiring the study protocols to be registered before being submitted to the journal. The remaining 5% recommended registration or required authors to register specific aspect of the study (e.g. new species taxa in *Mycobank* or *Zoobak*).
  ItAs of journals belonging to Health Sciences and Life Sciences, or those published by medium or large publishers, or registered in the *DOAJ*, were more likely, while those belonging to Arts & Humanities, and Physical Sciences were less likely to mention registration.
15. Replication: ItAs of 3% of journals promoted and accepted for publication replication studies, while 21% specified that study methods and analysis should be written in a way that facilitates replication. Scopes of 6 journals in our sample stressed that studies should be written in a way that facilitates replication, and only one specified accepting replication studies (of the 7, all but 1 also mentioned the same information in their ItAs).
  ItAs of journals belonging to Health Sciences or Life Sciences, or those published by medium or large publishers, or registered in the *DOAJ*, were more likely to mention replication.
16. Reporting Guidelines: ItAs of 13% of journals recommended the use of reporting guidelines for reporting of studies, while 2% required it. Details per guideline are presented in S1 Table, no journals required authors to check the *Equator* network, while 5% recommended it.
  ItAs of journals belonging to Health Sciences or Life Sciences, or those published by large publishers, were more likely to mention reporting guidelines.
17. Shared Authorship: ItAs of 2% of journals addressed shared/equal contributorship on a paper, with 1% allowing for joint first or senior authorship, 0.3% allowing for two co-authors be specified as having contributed equally without specifying if they need to be first or last/senior authors, 0.9% of the multidisciplinary journals allowing two or more authors to be designated as having equal contributorship, 0.1% allowing shared authorship, but not specifying the number or the status/seniority of the authors, and 0.1% stated that in general, no more than two shared first and/or senior authorships could be specified.
  ItAs of journals published by large publishers were more likely to mention shared/equal contributorship.
18. Statistics: Specific statistical reporting requirements were only occasionally addressed in the ItAs: 0.1% recommended reporting of Bayes factor(s), 3% recommended reporting of confidence intervals (CI) and 0.3% required CI be reported, 3% recommended reporting effect size, and 0.4% recommended reporting of sample size calculation.
  ItAs of journals belonging to Health Sciences or Life Sciences were more likely to mention confidence intervals, while those published by large publishers and with higher *SNIP* values were more likely to mention reporting of Bayes factor(s). Effect size was more likely to be mentioned in journals belonging to Health Sciences.
19. TOP Guidelines: ItAs of 1.7% of journals endorsed the TOP Guidelines of which almost all were published by *Emerald Group Publishing* (a medium publisher in our study). Subsequently, in the regression analysis journals published by medium publishers were more likely to mention the TOP Guidelines.

## Discussion

Our study, based on a representative sample of journals indexed in *Scopus*, showed that journals’ *Instructions to Authors* (ItAs) addressed on average only 4 out of the 19 transparency in reporting and research integrity topics we explored. Most commonly addressed were conflicts of interest, in 63% of journals, peer review type, in 52%, and plagiarism in 46%, with all other topics addressed in less than a third of journals. While our study was not designed to explain the reasons behind such low coverage of these topics in ItAs, previous research has demonstrated the editors’ reluctance to address cases of scientific misconduct and publication errors,[29, 30] as well as to implement prevention policies.[31] Our study has also found differences between scientific disciplines, with the Health Sciences and Life Science journals being more likely to cover many of the topics in their ItAs, while those of Arts & Humanities being least likely to do so. This finding is consistent with previous studies,[26, 32] and may stem from the major differences between the fields. For instance in Arts & Humanities the number of authors is rarely more than one or two per paper,[33] what constitutes as data or methods is often quite different from other sciences,[34] ethics appraisals for the studies are usually not undertaken,[35] structured reporting with standardized subsection titles is less common,[36] and books remain the major publication medium.[3, 37, 38] Furthermore, *ICMJE recommendations*, have probably only a limited applicability outside Health Sciences or Life Sciences. Finally, meta-research into scientific publishing and peer review has been led by the Health Sciences,[39, 40] as was development of multiple reporting guidelines,[41] while the Physical Sciences have been the forerunners of manuscript sharing on preprint servers.[42]

We also showed that the journals registered in the *Directory of Open Access Journals* database, as well as those published by medium or large publishers, were more likely to cover more of the transparency in reporting and research integrity topics in their ItAs. With more than 43,000 scientific journals worldwide[3] and the complexities of reporting recommendations for each specific study type,[41] differences between disciplines, and various ways the publishing process has been manipulated or abused,[43] it seems evident that journals and editors may benefit from publisher, societal or editor associations when drafting or updating their instructions, and implementing procedures that ensure compliance with requirements stated within them. Given that the coverage of topics in ItAs is increasing over time,[20] perhaps it is time that a uniform ItA which would cover all of these topics, akin to the Health Sciences specific *ICMJE Recommendations*, are produced that could then be adapted to specific needs of individual journals and disciplines. Alternatively, calls could be made to expand already existing guidelines (e.g. TOP guidelines) or to create complimentary ones that would cover all these topics. Additionally, as most scientific publishing today is predominantly handled through online submission systems, ItAs might also benefit from moving away from a (downloadable) document form to full integration within those systems, where each topic is explained, automatically checked and even converted to a specific journal-required form as the manuscript (or study protocol) is being submitted.

Finally, we have also shown that *Source Normalized Impact per Paper* (*SNIP*), i.e. citation metrics, were positively associated with mentioning of data sharing, image manipulation, and errata in ItAs. This is consistent with previous research, which indicated that top journals had more retractions and retraction policies, due to either their visibility and scrutiny or the willingness of authors to cut corners in order to publish in them.[44, 45]

Aside the low coverage of transparency in reporting and research integrity topics, it was interesting to discover that none of the websites of journals we analysed indicated which versions of the *ItAs* are currently on their websites, what (and why) were the changes from previous versions, and where previous versions could be found. This, akin to recent polemics on peer review, [11, 46, 47] perhaps further indicates that journals processes are not being scrutinized in the same way that publications published in those journals are. Furthermore, only 30% (38 out of 125) journals replied to our inquiries about their ItAs, also confirming previous finding of increased difficulty in engaging with editors or publishers regarding specific questions authors or researchers may have.[30]

To the best of our knowledge, ours is the first study to analyse journals ItAs across multiple scientific disciplines and wide ranges of journal citation metrics, however it is not without limitations. Firstly, due to our background and interests, we have focused on transparency in reporting and research integrity topics, which have received increased attention in the Health Sciences.[48] Furthermore, previous research has shown that some of the requirements editors impose on authors are not always listed in ItAs,[49, 50] some requirements that are listed are not always found in the published articles,[51] and vice versa, some topics are covered even if not addressed in ItAs.[52] Additionally, some of the journals also had instructions for reviewers or editorial policies, and these may have covered some of the topics we were interested in, but as such were not the aim of our study. For example, as information on data sharing in ItAs could contain links to publisher’s or editorial policies, which were outside our scope, we could not assess how many journals require data availability statements to be included in published manuscripts. Such statements, especially when they include links to deposited datasets, have been associated with an increase in paper citations and data discoverability,[53, 54] and increase authors’ compliance with journal’s recommendations or requirements regarding data sharing.[55]

We also limited our research to journals that have been classified as belonging exclusively to one of the Arts & Humanities, Health Sciences, Life Sciences, Physical Sciences, Social Sciences, or those classified as publishing papers from all of those categories (Multidisciplinary) which constitute 70% of journals in *Scopus*, so we cannot make inferences regarding the journals that are classified as covering two or more disciplines, but still not classified as multidisciplinary. Lastly, we used machine-assistance in analysing ItAs, by extracting sentences that contained keywords specific for the topics, making it possible we missed some of the information written. However, the iterative process of designing regular expressions and reading a number of ItAs in full makes such omissions unlikely.

In conclusion, our study showed that transparency in reporting and research integrity topics are insufficiently addressed in journals’ *Instructions to Authors*, leaving much to be desired in terms of what is asked of or recommended to authors. If journals wish to raise awareness of these topics and ensure compliance in addressing them, they could benefit from updating their ItAs and ensuring that requirements stated in ItAs match their practices. Furthermore, future research should try to determine barriers journals or editors face when implementing policy changes in their journals, and ways automated systems could reduce the burden of their work while ensuring compliance with specific journal or scholarly practices.

## Supporting information

S1 Table Percentages of journals covering transparency in reporting and research integrity topics in journals’ instructions to authors. *Our sample size was 835 journals (n). All analyses were performed in STATA (version 13) using sampling weights representing a total of 14,814 journals (N_w_).
(DOCX)

S2 Table Association (odds ratios from regression analysis) of transparency in reporting and research integrity topics addressed in instructions to authors of journals with: journals' Source Normalised Impact per Paper (SNIP) values, registration in the Directory of Open Access Journals (DOAJ), publishers’ category (medium or large sized publishers), and top scientific areas. Numbers in bold indicate found statistically positive associations and those in orange statistically negative associations. * For the regression analyses, reference categories were: 1) SNIP increase of 1; 2) Not registered in DOAJ; 3) Belonging to small publishers (defined as having only 1 journal in our sample form the same publisher); 4) Multidisciplinary Sciences journals.
(DOCX)
