# Supplementary material for: Journals’ instructions to authors: A cross-sectional study across scientific disciplines
Source: PLoS One. 2019 Sep 5;14(9):e0222157. doi: 10.1371/journal.pone.0222157 (PMC6728033; doi:10.1371/journal.pone.0222157)
Supplement: S1 Table — *Our sample size was 835 journals (n). All analyses were performed in STATA (version 13) using sampling weights representing a total of 14,814 journals (Nw). (DOCX) [file pone.0222157.s001.docx]

Supplementary Table 1. Percentages of journals covering transparency in reporting and research integrity topics in journals’ instructions to authors.

| **Topic** | **Journals (%, 95% CI, weighted*) belonging to** | | | | | | **Total  (n=835,**  **N_w_=14,814)** |
| --- | --- | --- | --- | --- | --- | --- | --- |
|  | **Arts & Humanities (n=132,**  **N_w_=1,052)** | **Health Sciences (n=153,**  **N_w_=3,966)** | **Life**  **Sciences (n=141,**  **N_w_=1,754)** | **Physical Sciences (n=162,**  **N_w_=4,586)** | **Social Sciences (n=153,**  **N_w_=3,350)** | **Multidisciplinary Sciences (n=94,**  **N_w_=106)** |  |
| **Conflicts of Interest** | 22 | 89 | 67 | 53 | 56 | 61 | 63 |
|  | (16-29) | (83-93) | (60-74) | (46-60) | (48-63) | (57-64) | (59-66) |
| Crossref Funder Registry | 0 | 0 | 5 | 1 | 1 | 0 | 1 |
|  |  |  | (3-9) | (0-4) | (0-5) |  | (1-2) |
| **COPE** | 6 | 31 | 25 | 21 | 26 | 15 | 24 |
|  | (3-11) | (24-38) | (19-32) | (15-28) | (20-34) | (13-18) | (21-27) |
| **Data Sharing** | 3 | 32 | 35 | 33 | 26 | 32 | 29 |
|  | (1-7) | (26-40) | (28-43) | (26-40) | (20-34) | (29-36) | (26-33) |
| Dryad | 1 | 3 | 9 | 6 | 5 | 15 | 5 |
|  | (0-5) | (1-8) | (6-15) | (3-10) | (2-9) | (12-17) | (4-7) |
| Figshare | 7 | 11 | 11 | 10 | 14 | 18 | 11 |
|  | (4-12) | (7-16) | (7-16) | (6-15) | (9-20) | (15-20) | (9-13) |
| Re3data | 0 | 1 | 1 | 2 | 1 | 3 | 1 |
|  |  | (0-5) | (0-5) | (1-6) | (0-5) | (2-4) | (1-3) |
| **Errata** | 5 | 39 | 38 | 37 | 20 | 24 | 31 |
|  | (2-9) | (31-47) | (31-45) | (30-44) | (15-27) | (21-27) | (28-35) |
| **Ethics Approval** | 0 | 74 | 33 | 7 | 13 | 20 | 29 |
|  |  | (67-81) | (27-40) | (4-13) | (8-19) | (18-23) | (27-32) |
| **ICMJE** | 0 | 72 | 21 | 3 | 4 | 5 | 24 |
|  |  | (65-79) | (15-27) | (1-7) | (2-8) | (4-6) | (21-26) |
| **Image Manipulation** | 3 | 15 | 21 | 11 | 10 | 12 | 12 |
|  | (1-7) | (10-22) | (15-28) | (7-16) | (6-16) | (10-14) | (10-15) |
| **(La)Tex** | 14 | 29 | 35 | 64 | 33 | 46 | 41 |
|  | (9-20) | (23-37) | (28-43) | (57-71) | (26-41) | (42-49) | (37-44) |
| **Limitations** | 0 | 22 | 11 | 3 | 5 | 4 | 9 |
|  |  | (16-29) | (7-16) | (1-7) | (2-9) | (3-6) | (7-11) |
| **Null Results** | 0 | 5 | 3 | 0 | 3 | 1 | 2 |
|  |  | (2-10) | (1-7) |  | (1-7) | (0-2) | (1-4) |
| **ORCID** | 11 | 20 | 22 | 19 | 22 | 16 | 20 |
|  | (7-17) | (14-27) | (16-29) | (14-26) | (17-30) | (14-19) | (17-23) |
| **Peer Review Type** | 61 | 53 | 43 | 41 | 72 | 35 | 52 |
|  | (53-68) | (45-60) | (35-51) | (33-48) | (64-78) | (31-38) | (49-56) |
| **Plagiarism** | 20 | 44 | 55 | 52 | 46 | 34 | 46 |
|  | (15-28) | (36-52) | (47-62) | (45-60) | (38-53) | (31-37) | (43-50) |
| **Preprint** | 11 | 26 | 32 | 25 | 14 | 18 | 22 |
|  | (7-17) | (20-34) | (25-39) | (19-32) | (10-21) | (16-21) | (19-26) |
| **Registration** | 2 | 38 | 22 | 2 | 3 | 10 | 15 |
|  | (0-5) | (31-46) | (16-29) | (1-6) | (1-8) | (8-12) | (12-17) |
| **Replication** | 0 | 30 | 35 | 24 | 19 | 21 | 24 |
|  |  | (24-38) | (28-43) | (18-31) | (14-26) | (18-24) | (21-28) |
| **Reporting Guidelines** | 2 | 36 | 19 | 4 | 9 | 6 | 15 |
|  | (0-5) | (29-44) | (14-26) | (2-8) | (5-14) | (5-8) | (13-18) |
| ARRIVE | 0 | 14 | 18 | 2 | 2 | 3 | 7 |
|  |  | (9-20) | (13-25) | (1-6) | (1-6) | (2-5) | (5-9) |
| recommend | 0 | 14 | 17 | 2 | 2 | 2 | 7 |
|  |  | (9-20) | (12-24) | (1-6) | (1-6) | (1-4) | (5-9) |
| require | 0 | 0 | 1 | 0 | 0 | 1 | 0 |
|  |  |  | (0-4) |  |  | (0-2) | (0-0) |
| CONSORT | 0 | 27 | 8 | 0 | 3 | 3 | 9 |
|  |  | (21-35) | (5-13) |  | (1-7) | (2-4) | (7-11) |
| recommend | 0 | 23 | 6 | 0 | 1 | 0 | 7 |
|  |  | (17-30) | (4-11) |  | (0-5) |  | (5-9) |
| require | 0 | 5 | 1 | 0 | 2 | 3 | 2 |
|  |  | (2-9) | (0-5) |  | (1-6) | (2-4) | (1-3) |
| PRISMA | 0 | 19 | 8 | 1 | 2 | 1 | 7 |
|  |  | (13-25) | (4-13) | (0-4) | (1-6) | (0-2) | (5-9) |
| recommend | 0 | 17 | 7 | 1 | 1 | 0 | 6 |
|  |  | (12-24) | (4-12) | (0-4) | (0-5) |  | (4-8) |
| require | 0 | 1 | 1 | 0 | 1 | 1 | 1 |
|  |  | (0-5) | (0-6) |  | (0-5) | (0-2) | (0-2) |
| STROBE | 0 | 13 | 6 | 0 | 1 | 1 | 4 |
|  |  | (8-19) | (3-11) |  | (0-5) | (0-2) | (3-6) |
| recommend | 0 | 11 | 5 | 0 | 1 | 1 | 4 |
|  |  | (7-17) | (2-10) |  | (0-5) | (0-2) | (3-6) |
| require | 0 | 1 | 1 | 0 | 0 | 0 | 0 |
|  |  | (0-5) | (0-6) |  |  |  | (0-1) |
| EQUATOR Network | 0 | 14 | 7 | 0 | 3 | 2 | 5 |
|  |  | (9-20) | (4-13) |  | (1-7) | (1-3) | (4-7) |
| **Shared Authorship** | 0 | 0 | 8 | 1 | 3 | 3 | 2 |
|  |  |  | (5-14) | (0-5) | (1-7) | (2-5) | (1-3) |
| **Statistics** | 1 | 16 | 6 | 0 | 2 | 2 | 6 |
|  | (0-5) | (11-23) | (3-11) |  | (1-6) | (1-4) | (4-8) |
| Bayesian statistics | 1 | 0 | 0 | 0 | 0 | 1 | 0 |
|  | (0-5) |  |  |  |  | (0-2) | (0-0) |
| Confidence  Intervals | 0 | 10 | 4 | 0 | 1 | 1 | 3 |
|  |  | (6-16) | (2-9) |  | (0-4) | (1-3) | (2-5) |
| Effect Size | 1 | 7 | 2 | 0 | 2 | 0 | 3 |
|  | (0-5) | (4-12) | (1-7) |  | (1-6) |  | (2-4) |
| Sample Size | 0 | 1 | 2 | 0 | 0 | 0 | 0 |
|  |  | (0-5) | (0-6) |  |  |  | (0-1) |
| **TOP Guidelines** | 0 | 1 | 1 | 1 | 4 | 1 | 2 |
|  |  | (0-5) | (0-4) | (0-5) | (2-8) | (0-2) | (1-3) |

*Our sample size was 835 journals (n). All analyses were performed in STATA (version 13) using sampling weights representing a total of 14,814 journals (N_w_).
