# Supplementary material for: Journals’ instructions to authors: A cross-sectional study across scientific disciplines
Source: PLoS One. 2019 Sep 5;14(9):e0222157. doi: 10.1371/journal.pone.0222157 (PMC6728033; doi:10.1371/journal.pone.0222157)
Supplement: S2 Table — Numbers in bold indicate found statistically positive associations and those in orange statistically negative associations. * For the regression analyses, reference categories were: 1) SNIP increase of 1; 2) Not registered in DOAJ; 3) Belonging to small publishers (defined as having only 1 journal in our sample form the same publisher); 4) Multidisciplinary Sciences journals. (DOCX) [file pone.0222157.s002.docx]

Supplementary Table 2. Association (odds ratios from regression analysis) of transparency in reporting and research integrity topics addressed in instructions to authors of journals with: journals' *Source Normalised Impact per Paper (SNIP)* values, registration in the *Directory of Open Access Journals (DOAJ)*, publishers’ category (medium or large sized publishers), and top scientific areas. Numbers in bold indicate found statistically positive associations and those in orange statistically negative associations.

| **Topic** | **OR (95% CI)** | | | | | | | | | **Constant (log odds)** |
| --- | --- | --- | --- | --- | --- | --- | --- | --- | --- | --- |
|  | **2016 SNIP** | **Publisher size*** | | **Registered in DOAJ database*** | **Scientific area*** | | | | |  |
|  |  | **Medium** | **Large** |  | **Arts and Humanities** | **Health Sciences** | **Life Sciences** | **Physical Sciences** | **Social Sciences** |  |
| **Conflict of Interest** | 1.3 (0.9-1.8) | **2.6 (1.6-4.1)** | **9.6 (5.9-15.6)** | **2.2 (1.3-3.6)** | 0.1 (0.1-0.2) | **4.6 (2.6-8.1)** | 0.9 (0.6-1.4) | 0.5 (0.3-0.7) | 0.6 (0.4-0.8) | 0.6 (0.4-0.9) |
| Crossref  Funder  Registry | 0.9 (0.6-1.4) | 1.0 (-) | **5.7 (1.2-26.7)** | 0.5 (0.1-3.4) | 1.0 (-) | 1.0 (-) | 4.2 (0.9-20.2) | 0.5 (0.0-6.3) | 1.0 (-) | 0.0 (0.0-0.0) |
| **COPE** | 1.0 (0.8-1.3) | **1.8 (1.1-2.9)** | **2.0 (1.3-3.2)** | 1.0 (0.6-1.6) | 0.4 (0.2-0.7) | **2.2 (1.5-3.4)** | **1.7 (1.1-2.5)** | 1.3 (0.8-2.0) | **1.7 (1.1-2.6)** | 0.1 (0.1-0.2) |
| **Data Sharing** | **1.5 (1.1-2.1)** | 1.7 (1.0-3.0) | **7.8 (4.8-12.7)** | 1.7 (1.0-2.9) | 0.1 (0.0-0.2) | 0.7 (0.5-1.1) | 0.7 (0.5-1.1) | 0.7 (0.5-1.1) | 0.5 (0.3-0.8) | 0.2 (0.1-0.3) |
| Dryad | **1.2 (0.9-1.6)** | 1.0 (0.3-3.7) | **3.7 (1.5-9.0)** | **2.4 (1.1-5.6)** | 0.1 (0.0-0.4) | 0.2 (0.1-0.4) | 0.4 (0.2-0.8) | 0.3 (0.1-0.6) | 0.3 (0.1-0.6) | 0.1 (0.0-0.2) |
| Figshare | 0.9 (0.6-1.4) | 0.8 (0.2-2.8) | **15.4 (6.3-37.4)** | **5.4 (2.6-11.2)** | 0.3 (0.2-0.7) | 0.3 (0.1-0.5) | 0.2 (0.1-0.5) | 0.4 (0.2-0.7) | 0.6 (0.3-1.1) | 0.1 (0.0-0.1) |
| Re3data | **2.5 (1.4-4.5)** | **30.6 (2.2-428)** | 3.8 (0.2-63.3) | **230.5 (43.9-1210.2)** | 1.0 (-) | 0.5 (0.1-2.3) | 0.7 (0.2-2.5) | 4.3 (0.9-20.6) | 4 (0.3-55.1) | 0.0 (0.0-0.0) |
| **Errata** | **1.6 (1.1-2.2)** | 0.9 (0.5-1.4) | **3.1 (2.0-4.7)** | 1.5 (0.9-2.5) | 0.2 (0.1-0.4) | **1.9 (1.3-2.8)** | **1.5 (1.0-2.3)** | **1.7 (1.1-2.5)** | 0.7 (0.4-1.1) | 0.1 (0.1-0.2) |
| **Ethics Approval** | 0.9 (0.6-1.2) | 1.9 (1.0-3.6) | **3.1 (1.9-5.1)** | **2.3 (1.4-3.9)** | 1.0 (-) | **11.4 (7.5-17.4)** | **1.7 (1.2-2.5)** | 0.3 (0.2-0.6) | 0.6 (0.3-1.0) | 0.1 (0.1-0.2) |
| **ICMJE** | 1.2 (0.9-1.5) | 1.4 (0.7-3.0) | 1.0 (0.5-1.9) | **2.8 (1.4-5.5)** | 1.0 (-) | **66.8 (39.3-113.4)** | **5.7 (3.4-9.4)** | 0.8 (0.3-2.2) | 1.1 (0.4-2.6) | 0.0 (0.0-0.0) |
| **Image Manipulation** | **1.4 (1.0-1.9)** | 1.0 (0.5-2.1) | **2.3 (1.3-4.2)** | 1.6 (0.9-3.0) | 0.3 (0.1-0.8) | 1.3 (0.7-2.2) | **1.6 (1.0-2.7)** | 0.9 (0.5-1.5) | 0.8 (0.4-1.5) | 0.1 (0.0-0.1) |
| **Latex** | 1.2 (0.9-1.5) | **2.4 (1.5-3.8)** | **5.9 (3.8-9.2)** | 1 (0.6-1.6) | 0.1 (0.1-0.2) | 0.3 (0.2-0.5) | 0.4 (0.3-0.6) | **1.7 (1.1-2.4)** | 0.4 (0.2-0.6) | 0.4 (0.3-0.6) |
| **Limitations** | 1.1 (0.7-1.7) | **2.8 (1.4-5.6)** | 0.9 (0.5-1.9) | 1.7 (0.9-3.5) | 1.0 (-) | **7.3 (4.2-12.6)** | **3.2 (1.7-6.1)** | 0.8 (0.3-2.0) | 1.2 (0.5-2.8) | 0.0 (0.0-0.0) |
| **Null Results** | 0.4 (0.1-1.3) | 1.3 (0.3-5.1) | 1.3 (0.3-5.1) | 1.7 (0.5-6.1) | 1.0 (-) | **5.1 (1.9-14.2)** | **3.1 (1.0-9.8)** | 1.0 (-) | **3.4 (1.0-10.8)** | 0.0 (0.0-0.0) |
| **ORCID** | 0.8 (0.6-1.0) | 1.2 (0.6-2.4) | **6.5 (3.7-11.3)** | 1.8 (1.0-3.2) | 0.5 (0.3-1.0) | 0.9 (0.5-1.5) | 1 (0.6-1.6) | 1 (0.6-1.7) | 1.2 (0.8-1.9) | 0.1 (0.1-0.2) |
| **Peer Review Type** | 1.0 (0.8-1.3) | 1.3 (0.8-1.9) | **2.9 (2.0-4.4)** | **3.2 (2.1-5.1)** | **3.8 (2.5-5.8)** | **2.0 (1.3-2.9)** | 1.2 (0.8-1.7) | 1.4 (1.0-2.0) | **5.7 (3.8-8.5)** | 0.3 (0.2-0.4) |
| **Plagiarism** | 1.0 (0.8-1.3) | **1.8 (1.2-2.8)** | **6.1 (4.0-9.2)** | **1.7 (1.1-2.6)** | 0.5 (0.3-0.7) | 1.1 (0.8-1.7) | **1.8 (1.2-2.6)** | **1.8 (1.3-2.7)** | 1.3 (0.9-2.0) | 0.3 (0.2-0.4) |
| **Preprint** | 1.4 (1.0-2.0) | 1.7 (0.9-3.3) | **9 (5.3-15.2)** | 1.4 (0.8-2.5) | 0.5 (0.3-1) | 1.1 (0.7-1.8) | 1.4 (0.9-2.2) | 1.0 (0.6-1.7) | 0.5 (0.3-0.8) | 0.1 (0.0-0.1) |
| **Registration** | 1.0 (0.7-1.4) | **3.1 (1.6-6.2)** | **3.9 (2.0-7.6)** | **4.5 (2.5-8.1)** | 0.2 (0.1-0.8) | **6.1 (3.9-9.6)** | **2.5 (1.5-4.1)** | 0.3 (0.1-0.7) | 0.4 (0.2-1.1) | 0.0 (0.0-0.1) |
| **Replication** | 1.3 (1.0-1.8) | **2.2 (1.3-3.7)** | **3.0 (1.9-4.8)** | **2.1 (1.3-3.4)** | 1.0 (-) | **1.5 (1.0-2.3)** | **1.8 (1.2-2.7)** | 1.2 (0.8-1.8) | 0.9 (0.6-1.4) | 0.1 (0.1-0.2) |
| **Reporting Guidelines** | 1.3 (1.0-1.7) | 1.3 (0.7-2.6) | **1.8 (1.0-3.2)** | 1.8 (1.0-3.1) | 0.3 (0.1-1.2) | **9.2 (5.7-14.6)** | **3.4 (2.1-5.6)** | 0.6 (0.3-1.5) | 1.5 (0.8-2.9) | 0.0 (0.0-0.1) |
| ARRIVE | 1.1 (0.8-1.5) | 1.6 (0.5-5.0) | **8.2 (3.3-19.9)** | **3.2 (1.5-6.9)** | 1.0 (-) | **4.2 (2.1-8.2)** | **5.4 (2.9-10.2)** | 0.6 (0.2-2.0) | 0.6 (0.2-2.1) | 0.0 (0.0-0.0) |
| CONSORT | **1.4 (1.0-2.0)** | 0.9 (0.4-2.2) | 1.4 (0.7-2.9) | **2.3 (1.2-4.4)** | 1.0 (-) | **15.4 (8.3-28.7)** | **2.8 (1.4-5.9)** | 1.0 (-) | 1.4 (0.6-3.8) | 0.0 (0.0-0.0) |
| PRISMA | **1.5 (1.0-2.3)** | 1.3 (0.5-3.6) | 1.7 (0.7-4.0) | **2.9 (1.4-6.3)** | 1.0 (-) | **32.1 (11.4-89.7)** | **9.6 (3.4-26.9)** | 0.9 (0.1-6.9) | 3.2 (0.8-12.8) | 0.0 (0.0-0.0) |
| STROBE | **1.7 (1.0-2.7)** | 1.2 (0.3-4.7) | **4.4 (1.3-14.3)** | **4.4 (1.7-10.9)** | 1.0 (-) | **23.7 (5.6-99.7)** | **6.8 (1.8-25.0)** | 1.0 (-) | 2.3 (0.4-13.8) | 0.0 (0.0-0.0) |
| EQUATOR | **1.5 (1.1-2.2)** | 1.3 (0.4-3.9) | **2.6 (1.0-6.4)** | **2.9 (1.3-6.6)** | 1.0 (-) | **10.8 (4.4-26.6)** | **3.9 (1.6-9.9)** | 1.0 (-) | 2.1 (0.6-6.9) | 0.0 (0.0-0.0) |
| **Shared Authorship** | 1.1 (0.8-1.6) | 2.8 (0.5-15.3) | **5.9 (1.6-21.7)** | 0.8 (0.1-5.0) | 1.0 (-) | 1.0 (-) | 2 (0.9-4.5) | 0.2 (0.0-1.3) | 0.5 (0.2-1.6) | 0.0 (0.0-0.1) |
| **Statistics** | 1.2 (0.8-1.8) | 1.0 (0.3-2.5) | 0.8 (0.3-1.8) | 1.2 (0.5-2.7) | 0.4 (0.1-2.7) | **9.2 (4.6-18.5)** | **2.9 (1.2-6.9)** | 1.0 (-) | 1.0 (0.3-3.5) | 0.0 (0.0-0.0) |
| Bayesian | **1.6 (1.0-2.4)** | 1.0 (-) | **14.6 (1.5-141.1)** | 1.0 (-) | 1.0 (0.0-28.9) | 1.0 (-) | 1.0 (-) | 1.0 (-) | 1.0 (-) | 0.0 (0.0-0.1) |
| Confidence  intervals | 0.9 (0.3-2.7) | 1.5 (0.5-4.5) | 0.5 (0.2-1.9) | 1.5 (0.6-3.9) | 1.0 (-) | **9.7 (4.0-23.6)** | **3.9 (1.4-10.9)** | 1.0 (-) | 0.6 (0.1-4.4) | 0.0 (0.0-0.0) |
| Effect Size | 1.4 (0.9-2.1) | 0.4 (0.1-2.2) | 1.1 (0.4-3.2) | 0.9 (0.3-3.1) | 0.4 (0.0-3.7) | **3.9 (1.1-14.1)** | 1.0 (0.2-4.9) | 1.0 (-) | 1.0 (-) | 0.0 (0.0-0.1) |
| Sample size | 1.5 (-) | 1.0 (-) | 0.2 (-) | 1.0 (-) | 1.0 (-) | 0.5 (-) | 1.0 (-) | 1.0 (-) | 1.0 (-) | 0 (-) |
| **TOP Guidelines** | 0.4 (0.2-1.1) | **37.6 (5.3-268.7)** | 1.0 (-) | 0.3 (0.0-2.2) | 1.0 (-) | 1.3 (0.2-8.3) | 1.3 (0.1-15.8) | 1.2 (0.2-7.1) | 3.4 (0.8-14.3) | 0.0 (0.0-0.0) |

***** For the regression analyses, reference categories were: 1) SNIP increase of 1; 2) Not registered in DOAJ; 3) Belonging to small publishers (defined as having only 1 journal in our sample form the same publisher); 4) Multidisciplinary Sciences journals.
